# ICoN: integration using co-attention across biological networks

**DOI:** 10.1093/bioadv/vbae182

**Published:** 2024-11-22

**Authors:** Nure Tasnina, T M Murali

**Affiliations:** Department of Computer Science, Virginia Tech, Blacksburg, VA 24061, United States; Department of Computer Science, Virginia Tech, Blacksburg, VA 24061, United States

## Abstract

**Motivation:**

Molecular interaction networks are powerful tools for studying cellular functions. Integrating diverse types of networks enhances performance in downstream tasks such as gene module detection and protein function prediction. The challenge lies in extracting meaningful protein feature representations due to varying levels of sparsity and noise across these heterogeneous networks.

**Results:**

We propose ICoN, a novel unsupervised graph neural network model that takes multiple protein–protein association networks as inputs and generates a feature representation for each protein that integrates the topological information from all the networks. A key contribution of ICoN is exploiting a mechanism called “co-attention” that enables cross-network communication during training. The model also incorporates a denoising training technique, introducing perturbations to each input network and training the model to reconstruct the original network from its corrupted version. Our experimental results demonstrate that ICoN surpasses individual networks across three downstream tasks: gene module detection, gene coannotation prediction, and protein function prediction. Compared to existing unsupervised network integration models, ICoN exhibits superior performance across the majority of downstream tasks and shows enhanced robustness against noise. This work introduces a promising approach for effectively integrating diverse protein–protein association networks, aiming to achieve a biologically meaningful representation of proteins.

**Availability and implementation:**

The ICoN software is available under the GNU Public License v3 at https://github.com/Murali-group/ICoN.

## 1 Introduction

The emergence of high-throughput experimental techniques has led to the development of extensive protein–protein association networks. In such a network, each node is a protein and an edge between two nodes represents a certain type of association, e.g., physical binding, shared cellular function, or correlated expression. In principle, each type of interaction yields a separate network (Costanzo *et al.* 2016, Oughtred *et al.* 2021). Since different types of networks provide heterogeneous and complementary biological information, integrating them into a common representation allows for improved performance over using a single data source in tackling questions such as protein function prediction and gene module detection (Wass *et al.* 2012, Forster *et al.* 2022). However, this integration is not trivial due to the diversity of experimental methods (Forster *et al.* 2022). Moreover, networks may include false positive interactions, e.g., by retaining interactions that occur due to nonspecific binding in tandem affinity purification coupled with mass-spectrometry analysis (Sun *et al.* 2013). Interactions may also be missing due to a lack of resources. Early techniques for network integration relied on supervision using protein function annotations as labels (Huttenhower *et al.* 2006, Mostafavi *et al.* 2008). However, the performance of these models is contingent on the availability and quality of the provided labels. Moreover, these models may not generalize well to other downstream tasks that they were not trained on.

A recent line of research in network integration is to obtain a feature representation (or embedding) for each protein that captures topological information from all input networks. Four methods, Mashup (Cho *et al.* 2016), deepNF (Gligorijević *et al.* 2018), BIONIC (Forster *et al.* 2022), and BERTWalk (Nasser and Sharan 2023) have presented unsupervised approaches to generate protein embeddings. They used these embeddings to obtain promising performance in downstream tasks, e.g., protein function prediction and module detection. Mashup (Cho *et al.* 2016) performed random walks with restart (RWR) on each input network, yielding network-specific “diffusion states” for each node. These states reflect the probability of reaching every node at the steady state. Mashup then solved an optimization problem that computes low-dimensional vector representation (or embeddings) of nodes that best approximate the diffusion states according to a multinomial logistic model. deepNF (Gligorijević *et al.* 2018) proposed a multimodal deep autoencoder to learn embedding for a protein. It first created a low-dimensional representation of each protein for each network by executing RWR for a specified number of steps, followed by constructing a Positive Pointwise Mutual Information matrix. Subsequently, deepNF concatenated these representations for each protein from multiple networks and passed the combined vectors through an autoencoder to construct the final embeddings.

BIONIC (Forster *et al.* 2022) processed the input networks independently through multiple layers of graph attention networks (GAT) (Veličković *et al.* 2018) to obtain network-specific protein embeddings. Consequently, BIONIC averaged the network-specific embeddings of each protein. Both deepNF and BIONIC trained their neural networks by minimizing the reconstruction loss. BERTWalk (Nasser and Sharan 2023) transformed each input network into a collection of node sequences where each sequence represents a path obtained from a random walk of length 10. Treating these node sequences as sentences, BERTWalk constructed a corpus by amalgamating sentences from all input networks. Subsequently, the authors employed a masked language modeling technique as used by BERT (Devlin *et al.* 2019), training it to identify masked tokens (nodes) within sentences. Finally, they extracted a representation of the proteins from the embedding layer of their model.

Each of these methods computes initial embeddings independently for each network. These embeddings encode the topological information for each network. They do not allow this information to be shared across the input networks during training. We hypothesized that a model that facilitates inter-network communication during training may learn a representation of proteins that yields superior performance in downstream tasks. In this context, we embraced the notion of multi-modal alignment that is utilized in vision-language models (Lu *et al.* 2019, Tan and Bansal 2019) and has demonstrated potential in the biological applications such as drug-target annotation (Huang *et al.* 2022) and cancer driver gene prediction (Zhao *et al.* 2022). This approach facilitates integration across diverse data types such as image, text, and video by leveraging shared attention mechanisms, i.e., co-attention across modality-specific transformer encoders (Lu *et al.* 2019). We introduce this idea in the context of unsupervised network integration by developing a new co-attention mechanism that aligns multiple topological contexts of a single protein originating from heterogeneous networks to obtain an embedding for each protein.

In an attempt to make the integration model robust to noise, we adopt a training technique inspired by denoising autoencoders (DA) (Vincent *et al.* 2008, Cao *et al.* 2016; Gidaris and Komodakis 2019). In principle, a DA trains a model to learn the original inputs from corrupted or noisy data by deliberately introducing noise into the input during the training process.

### Our contributions

We propose ICoN (“**I**ntegration using **Co**-attention across Biological **N**etworks”), a novel co-attention-based, denoising, unsupervised graph neural network model that takes multiple protein association networks as inputs and generates a feature representation for each protein. A traditional GAT model generates a node’s embeddings by aggregating features from its neighbors in only one network, learning varying degrees of priority or attention for each neighbor. In contrast, ICoN learns the attention given to other nodes by considering the topological context of that node across *all* input networks. This concept of co-attention permits cross-network communication during training. Furthermore, we adopt a denoising training technique where we introduce perturbation to each input network and train the model to reconstruct the original one from the corrupted version. To the best of our knowledge, this method has not been previously employed in the context of network integration.

### Our results

We evaluate ICoN using the evaluation framework developed by BIONIC that includes three downstream tasks (gene module detection, gene coannotation prediction, and gene function prediction) and three benchmark datasets. We demonstrate that the embeddings produced by ICoN outperform those of individual networks across all downstream tasks. In the majority of downstream tasks, ICoN outranked existing unsupervised network integration models. We also demonstrate that the co-attention coefficients learned by ICoN can be interpreted meaningfully in the context of the noise we artificially add during training. Furthermore, we illustrate ICoN’s superior robustness toward noise compared to BIONIC.

## 2 Methods

For each network, we consider the union of the nodes across all the input networks, i.e. a node not originally in a network will now appear as disconnected in this network. The initial feature for every node is a one-hot encoding, i.e. a vector whose length is equal to the total number of nodes, and the index denoting the corresponding node contains 1 while the rest of the indices have the value 0.

### 2.1 Model architecture

**Figure 1.**
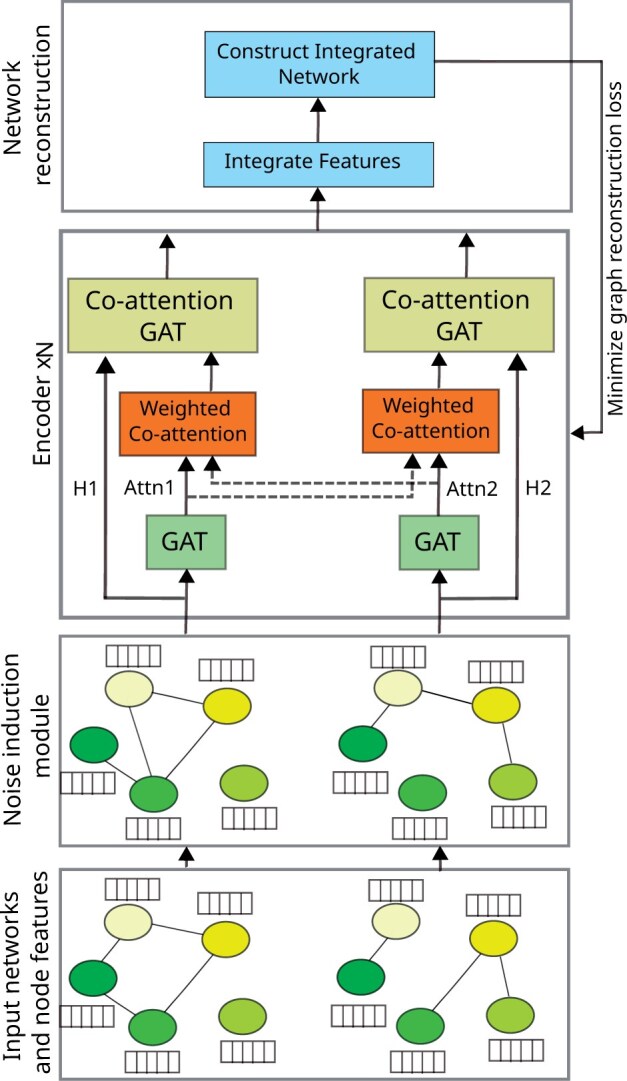
Architecture of ICoN. Attn1 and Attn2 are the learned attention matrices from the corresponding GAT layers, whereas H1 and H2 are the feature representations of nodes from the corresponding networks.

There are three principal blocks in ICoN (Fig. 1): (i) the noise induction module that introduces a specific amount of noise to each of the input networks before inputting them to the subsequent encoder module. (ii) the encoder module computes the embedding of each protein allowing cross-network communication among input networks. (iii) the “network reconstruction module constructs an integrated network after learning embedding for each protein.

#### 2.1.1 Noise induction module

We introduce noise to each of the input networks by randomly removing a fraction (dictated by hyperparameter) of existing edges and incorporating the same number of new edges, selected uniformly at random from the set of all node pairs not connected by an edge in the network.

#### 2.1.2 Encoder module

To reduce memory usage and speed up training, we map the initial one-hot encoding feature to a lower-dimensional space using a learnable linear transformation layer.

We then pass this low-dimensional feature to multiple (*N*) stacked blocks of similar architecture. Each block has two main components: (i) a traditional GAT layer that computes the attention of each node toward its neighboring nodes and (ii) a novel co-attention-based GAT that incorporates the attention computed by the first GAT layer for individual input networks and utilizes that to learn a cross-network aware representation of each protein.

##### GAT

Given the adjacency matrix *A^m^* of a network *m*, a traditional GAT computes an embedding for a node by aggregating the features of its neighboring nodes but after giving different priorities or “attention” values to each neighbor. We compute the attention $\alpha_{ij}^{m}$ that node *i* gives to node *j* in network *m* as follows:

$$\alpha_{ij}^{m}\propto A_{ij}^{m}\;\text{exp}(\sigma (a^{m}{}^{T}(W^{m}h_{i}^{m}||W^{m}h_{j}^{m})))$$

Here, $A_{ij}^{m}>0$ iff *i* and *j* are connected by an edge in network *m*, $W^{m}\in R^{d\prime \times d}$ is a network-specific learnable weight matrix where *d* and d′ are input and output feature dimensions respectively for the GAT layer, $a\in R^{2d\prime }$ is a vector of learnable attention coefficients. *σ* is a nonlinear activation function, e.g. LeakyRelu, *h_i_* is the feature vector of node *i* that is input to the GAT layer, and || denotes concatenation operation. The constant of proportionality in the equation ensures that the sum of $\alpha_{ij}^{m}$ over all the neighbors of node *i* equals 1. It is noteworthy that we employ neighbor sampling (Hamilton *et al.* 2017) by selecting a subset of neighbors while feature aggregation to reduce computational expense (Supplementary Section S1.1).

##### Co-attention GAT

For network *m*, we compute an *n *×* n* co-attention matrix *χ^m^*, which is a weighted average of the already computed attention from all the input networks in the previous GAT layers. Specifically, we compute $\chi_{ij}^{m}$, the co-attention that node *i* gives to node *j* as follows:

$$\chi_{ij}^{m}=\sum_{k=1}^{M}c_{k}^{m}\alpha_{ij}^{k}\;\text{where},\sum_{k=1}^{M}c_{k}^{m}=1$$

Here, *M* is the total number of input networks and the learnable *co-attention coefficient*, $c_{k}^{m}$ denotes the priority given by network *m* to (the attention computed by the GAT layer on) network *k*. Finally, we compute the embedding for node *i* in network *m* as follows:

$$h_{i}^{\prime m}=\sigma (\sum_{j\in N_{i}}\chi_{ij}^{m}W^{m}h_{j}^{m})$$

This new vector will be the input for the next GAT layer.

#### 2.1.3 Network reconstruction module

The encoder module generates separate embeddings of a protein for each of the input networks. The first step of the reconstruction module is to average these embeddings across all the networks to get the final embedding of a protein. To construct the integrated network, we utilize a simple dot product operation on the computed embeddings of pairs of nodes. Suppose, matrix *F* contains the embeddings of each node in the network. We compute the adjacency matrix $\hat{A}$ of the reconstructed network as follows: $\hat{A}=F.F^{T}$

##### Loss computation

We train ICoN to minimize the discrepancy between the reconstructed network and the original input networks (without noise). This strategy empowers our model to navigate through noisy networks to reach a denoised integrated network. We formulate the network reconstruction loss *L* as follows, where *n* is the total number of nodes and $||.|{|}_{F}$ is the Frobenius norm: $L=\frac{1}{n^{2}}\sum_{k=1}^{M}||(\hat{A}-A^{k})|{|}_{F}^{2}$

### 2.2 Datasets

We used the input networks used by BIONIC (Forster *et al.* 2022) and subsequently utilized by BERTWalk (Nasser and Sharan 2023). We briefly describe these networks in this section.

We ran ICoN on heterogeneous networks originating from diverse experiments for *Saccharomyces cerevisiae* and human. We integrated three baker’s yeast (i.e. *Saccharomyces cerevisiae*) networks: (i) a protein–protein interaction (PPI) network (2674 genes and 7075 interactions) obtained by tandem affinity purification followed by mass spectrometry (Krogan *et al.* 2006), (ii) a genetic interaction (GI) network (4529 genes and 33 056 interactions) constructed by calculating the pairwise Pearson correlation between genetic interaction profiles of genes (Costanzo *et al.* 2016), and (iii) a coexpression (COEX) network (1101 genes and 14 826 interactions) based on the Pearson correlation between transcriptional response profiles of deletion yeast strains (Hu *et al.* 2007) (Supplementary Section S1.2, Supplementary Table S5). We also integrated four human PPI networks emerging from diverse experimental approaches: (i) Rolland-14 (4301 genes and 13 940 interactions) (Rolland *et al.* 2014), (ii) Hein-15 (5380 genes and 27 349 interactions) (Hein *et al.* 2015), (iii) Huttlin-15 (7658 genes and 23 712 interactions) (Huttlin *et al.* 2015), and (iv) Huttlin-17 (10 945 genes and 56 471 interactions) (Huttlin *et al.* 2017) (Supplementary Section S1.2, Supplementary Table S5). This analysis permitted us to study ICoN’s generalizability across species and determine if there was any utility in integrating networks containing the same type of interactions (PPIs) but generated by different groups using complementary experimental techniques.

### 2.3 Evaluation

We have followed the evaluation pipeline for network integration introduced by BIONIC (Forster *et al.* 2022) and used by BERTWalk (Nasser and Sharan 2023). This framework consisted of three downstream tasks and three ground truth benchmark datasets.

#### 2.3.1 Downstream tasks

- gene module detection: Here, we have assessed the model’s ability to reproduce biological modules such as protein complexes and pathways by performing hierarchical clustering of nodes (based on embeddings) using a variety of distance metrics (Euclidean, cosine) and linkage methods (single, average, and complete). We calculated the adjusted mutual information (AMI) score between the derived clusters and the ground truth biological modules (from a specific benchmark) to measure their similarity while adjusting for the effect of agreement solely due to random chance. We optimized the clustering parameters (i.e., distance metric and linkage method) for each network integration method (and network) and reported the best AMI.
- gene coannotation prediction: The goal of this task was to evaluate how well a model preserves gene–gene relationships in its node embeddings. We predicted whether a pair of genes are annotated to the same term in a particular functional benchmark. Given the embeddings for a pair of genes, we computed the cosine similarity. We categorized gene pairs as positive (i.e., coannotated) or negative based on a predefined threshold. By varying this threshold, we computed the average precision.
- gene function prediction: This task sought to evaluate a model’s capability to generate embeddings that are effective in predicting known functional classes (such as membership to a particular protein complex or pathway) of a gene. Using gene embeddings as input features, we trained a support vector machine classifier (with a radial basis function kernel) to predict gene functions in a one-versus-all manner. We exploited 5-fold cross-validation to tune the classifier’s regularization and gamma parameters on the validation dataset. We evaluated the classifier on a randomized held-out set, consisting of 10% of the gene features not seen during training or validation, and reported the resulting classification accuracy. We repeated this process 5 times.

#### 2.3.2 Benchmark datasets

We used three datasets (the first two for yeast and the final one for *Homo sapiens*), each containing genes annotated to functional terms indicating their membership in certain protein complexes or pathways. (i) IntAct protein complexes (Orchard *et al.* 2014), (ii) Kyoto Encyclopedia of Genes and Genomes (KEGG) pathways (Kanehisa and Goto 2000), and (iii) CORUM complexes (Tsitsiridis *et al.* 2023). To create a coannotation benchmark from each of these three datasets, all gene pairs that share at least one functional annotation were considered to be positive pairs, while those lacking shared annotations were classified as negative pairs. The coannotation datasets for IntAct and KEGG contained 1,786 and 2,029 genes; 9,288 and 32,563 positive pairs; 1,584,717 and 2,024,843 negative pairs, respectively. The CORUM dataset for human contained 10,105 genes, 539,272 positive, and 50,901,158 negative pairs. We constructed the benchmarks for module detection by defining a module as a collection of genes annotated to the same functional term. Modules with single genes in them were removed as they were uninformative. The module detection benchmarks included 574, 107, and 3,575 modules for IntAct, KEGG, and CORUM respectively. Finally, we formed the benchmark for function prediction by considering the functional annotation of a gene as its class label. IntAct, KEGG, and CORUM consisted of 48, 53, and 296 functional classes, respectively. We also used a fourth ground truth dataset based on Gene Ontology (Ashburner *et al.* 2000) annotations for baker’s yeast. Due to the poor performance shown by every model on this dataset, we describe this dataset and the corresponding results in Supplementary Section S1.3.

## 3 Results and discussion

First, we assessed the performance of the integrated features obtained through ICoN against features from individual input networks, based on different downstream tasks across varied functional benchmarks (Section 3.1). Next, we performed a comparative analysis of ICoN with four unsupervised network integration methods: Mashup, deepNF, BIONIC, and BERTWalk (Section 3.2). We ran all algorithms (with the optimized hyperparameters (Supplementary Section S1.4)) five times, except for BERTWalk. Third, through an ablation study, we showcased the utility of two pivotal components of ICoN: co-attention GAT and noise induction (Section 3.3). Subsequently, we investigated the learned co-attention coefficients (Section 3.4). Finally, we analyzed the robustness of ICoN to noise present in the input networks (Section 3.5).

### 3.1 Improvement over individual input networks

**Figure 2.**
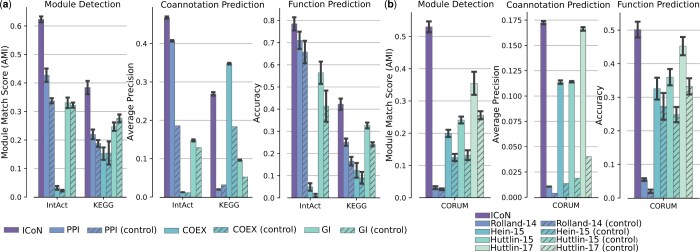
Evaluation of ICoN’s performance on downstream tasks. Comparison of the performance of ICoN (i.e., integration of all input networks) with (a) three individual yeast networks and (b) four individual human networks. Here, for each network, we have two entries, e.g., “PPI” and “PPI (control).” “PPI” denotes the results for the embedding generated by ICoN with only the PPI network as input and “PPI (control)” stands for the results obtained using the adjacency matrix-based features of the network. The height of each bar indicates the average of the corresponding score and the error bar shows the standard deviation.

In our first analysis, we compared the performance of ICoN with features derived from individual input networks. For each input network, we explored two methods of feature generation: (i) embeddings generated using ICoN with only the corresponding network as input, and (ii) interaction profiles of proteins (i.e., rows in the adjacency matrix of the network) as features. We found that the embeddings generated by ICoN upon integrating all three networks outperformed the ICoN-generated embeddings from each individual yeast network across all three tasks and functional benchmarks (Fig. 2a, Supplementary Table S2), except for coannotation prediction in the KEGG dataset. Furthermore, ICoN consistently outperformed features based on interaction profiles of individual yeast networks. The same result held for the four human PPI networks in the CORUM complexes benchmark (Fig. 2b, Supplementary Table S2). By using the embeddings generated by integrating all three networks with ICoN, we could identify modules that were not detected when using adjacency-based features of individual networks (Supplementary Section S1.5).

Note that the absolute performance of ICoN-based features changed from task to task and from one benchmark to another, as indicated by the different ranges of the *y*-axes in the plots (Fig. 2). Nevertheless, these results confirmed the efficacy of employing integrated features as opposed to relying on individual networks.

### 3.2 Improvement over existing unsupervised methods

**Figure 3.**
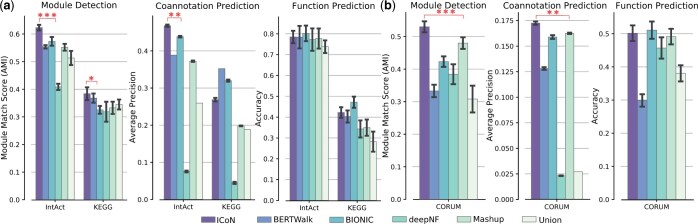
Comparison of ICoN and five unsupervised network integration methods on (a) three yeast networks and (b) four human networks. Here, the height of each bar indicates the average of the corresponding score, and the error bar shows the standard deviation. Single, double, and triple stars stand for significant *P*-values (<0.05, 0.01, and <0.001, respectively) of the improvement of ICoN over the second best model.

We compared ICoN to BERTWalk, BIONIC, deepNF, and Mashup and to Union, a naive approach where we computed the integrated network as the union of the node sets and edge sets across all the input networks. For the three yeast networks, ICoN outperformed all the methods in module detection across all the functional benchmarks (Fig. 3a, Supplementary Table S3). In each of these benchmarks, either BIONIC or BERTWalk emerged as the second-best model. ICoN obtained a significantly higher AMI compared to the second-best model (Mann-Whitney U test *p*-values of $3.98\times 10^{-18}$ in IntAct and 0.02 in KEGG). We also observed that ICoN surpassed each of the models in coannotation prediction in IntAct benchmark (Mann–Whitney *U* test *P*-values of 0.006). Finally, in function prediction, ICoN exhibited comparable performance, with no statistically significant difference, to the best-performing model, BIONIC in IntAct. However, in the KEGG benchmark, ICoN demonstrated inferior performance compared to BIONIC. For the human datasets, ICoN demonstrated a persistent pattern of excellence by outranking all five methods in module detection (Mann–Whitney *U* test *P*-value of $1.06\times 10^{-16}$) and coannotation prediction (Mann–Whitney *U* test *P*-value of 0.006) (Fig. 3b, Supplementary Table S3).

In summary, ICoN outperformed all the methods in five out of nine task-benchmark pairs while showing comparable performance to the best-performing model in two tasks. Additionally, it is noteworthy that in the two tasks where ICoN had the inferior performance, no single model emerged as the indisputable best performer.

### 3.3 Ablation study

**Figure 4.**
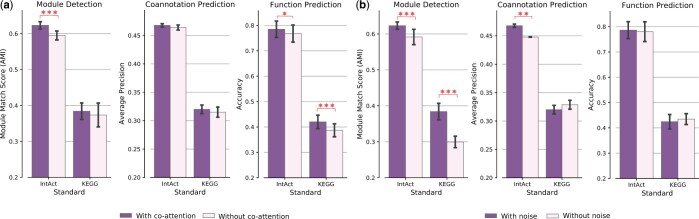
Results of ablation study on co-attention and noise induction. Single, double, and triple stars stand for significant *P*-values (<0.05, 0.01, and <0.001, respectively) of the improvement of ICoN having co-attention and noise.

We introduced co-attention in ICoN to facilitate the exchange of topological information across networks during training. To study the importance of co-attention, we conducted an experiment where we constrained ICoN to solely consider self-computed attention to aggregate features from its neighbors, thereby eliminating co-attention. We found that ICoN outperformed the restricted model on three out of six benchmark-tasks pairs (Mann–Whitney *U* test *P*-value < 0.05) (Fig. 4a, Supplementary Table S4).

In the second ablation study, we avoided the induction of noise into the input networks. Our findings indicate that the absence of noise adversely affects performance in three out of six benchmark-tasks pairs (Mann–Whitney *U* test *P*-value < 0.01) (Fig. 4b, Supplementary Table S4).

It is noteworthy that either co-attention or noise induction improved performance in five out of six benchmark-task pairs. Their complementarity enables ICoN to surpass existing network integration models.

### 3.4 Interpretation of the learned co-attention coefficients

**Figure 5.**
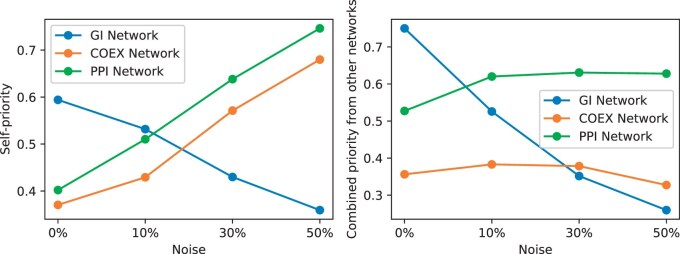
Variation in co-attention coefficient with noise (shown for the first layer of encoder module). Along the *x*-axis, we show the amount of noise introduced by the noise induction module. Along the *y*-axis we show (a) the amount of self-priority (i.e., learned co-attention coefficient) each network gives to itself and (b) the total amount of priority each network gets from other networks.

Here, we aimed to assess to what extent ICoN exploited its ability to share attention (i.e., co-attention) across networks. Additionally, we sought to investigate whether one network can assist another in mitigating the impact of noise through this shared attention mechanism. We first observed that for every value of induced noise (that we tested), each of the three yeast networks learned nonzero co-attention coefficients not for just itself but for other input networks as well (Fig. 5). As we increased noise, the priority given by the GI network to itself decreased substantially: from 0.59 at 0% noise to 0.36 at 50% noise (Fig. 5a). In contrast, the learned self-priority by the COEX and PPI networks increased with noise: from 0.37 at 0% noise to 0.68 at 50% noise for COEX and from 0.40 at 0% noise to 0.75 at 50% noise for PPI network. We also observed that as we increased noise, the priority GI network received from other networks decreased drastically: from 0.75 at 0% noise to 0.26 at 50% noise (Fig. 5b).

The GI network is the largest, encompassing 60% of the total number of edges, while the COEX and PPI networks contain 27% and 13% of the edges, respectively (Section 2.2). The larger the network, the more the number of true edges removed or false interactions introduced by the noise induction module. We surmised from Fig. 5 that the largest network mitigated the impact of this noise by assigning itself reduced self-priority. In contrast, the COEX and PPI networks refrained from adopting this approach, as diminishing self-priority would mean giving increased attention to the other networks, resulting in a higher influence of noise from them. This explanation also accounts for the decreased priority allocated to the GI network by the others as noise escalated.

Observing the low priority assigned to the GI network when the noise level was high, we analyzed the impact of excluding the GI network from integration (Supplementary Section S1.6). We observed that integrating only the COEX and PPI networks resulted in decreased performance across five out of six task-benchmark pairs. We concluded that although ICoN ignored the computed attention in GI network by assigning it a low priority, it still took advantage of the original, non-noisy GI network when minimizing the reconstruction loss.

### 3.5 Robustness to noise

A proposed network integration method should be robust to the presence of noise. To assess this property, we artificially introduced noise by dropping a certain percent of existing edges and then adding the same number of random edges to each original input network. Then we integrated the noisy networks employing ICoN and BIONIC. Unlike noise induction, where we introduce noise into the input networks and minimize the reconstruction loss on the original edges, here we measure the reconstruction loss with respect to the noisy network.

**Figure 6.**
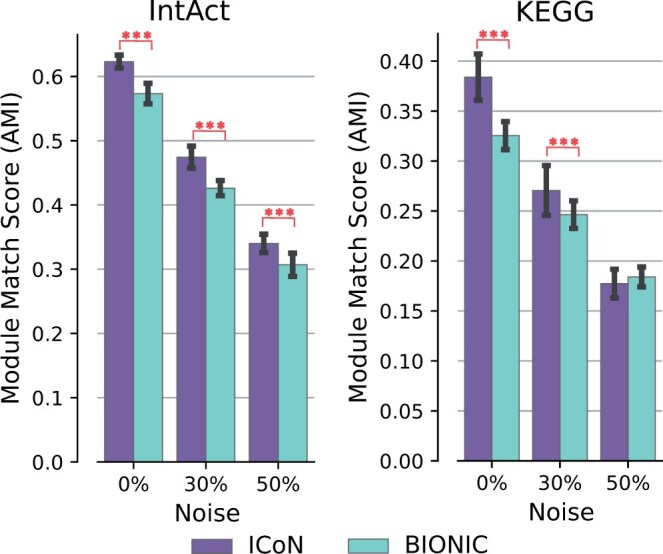
Comparison of ICoN and BIONIC regarding robustness to noisy inputs. Triple stars stand for significant *P*-values of <0.001 indicating superiority of ICoN over BIONIC.

In this analysis, we induced different levels of noise in three yeast networks. For the IntAct benchmark, we observed that ICoN maintained its superiority in module detection over BIONIC as the networks became noisier. For the KEGG benchmark, ICoN improved over BIONIC at the 0% and 30% noise levels but slightly underperformed compared to BIONIC at the 50% level. These results demonstrate ICoN’s robustness in handling noisy input networks (Fig. 6).

### 3.6 Scalability of ICoN

We determined ICoN’s scalability in terms of the requirement for graphics processing unit (GPU) memory and training time for each epoch (Supplementary Fig. S7). To assess the dependence of these values on the size of the input networks, we generated random, Erdös-Renyi networks with different numbers of nodes while keeping the average node degree as 30. We compared ICoN with BIONIC, the model that achieved the second-best performance. For networks with 20,000 nodes, ICoN could integrate up to five networks with GPU usage of 7.57 GB and average epoch time of 36.32 s, whereas BIONIC could integrate up to 10 such networks requiring substantially less time and memory. The higher memory consumption of ICoN is due to the need to store the learned feature matrices for all networks simultaneously at each intermediate GAT layer. The increased runtime of ICoN is due to the local indexing of nodes in GAT layers within individual networks. When sharing neighborhood information across networks, it was necessary to map node indices between networks, leading to this inefficiency.

## 4 Conclusions

In ICoN, we introduce a novel graph neural network architecture that facilitates attention sharing across networks to generate integrated protein embeddings. This unsupervised model, trained solely on network topology, exhibits superior generalization across the majority of downstream tasks compared to existing biological network integration models. Furthermore, ICoN demonstrates robustness against noisy networks.

Despite these advancements, there are several promising avenues for improvement. First, enhancing the model’s scalability is an important direction, particularly for handling larger, more complex biological networks. Additionally, developing techniques to biologically interpret the co-attention coefficients could provide valuable insights. Finally, while ICoN currently relies only on network topology, incorporating gene or protein features such as sequence, structure, and embeddings from large pre-trained models could potentially improve its performance.

## Supplementary Material

vbae182_Supplementary_Data

## Data Availability

The ICoN software and data are available under the GNU Public License v3 at https://github.com/Murali-group/ICoN.
