# Supplementary material for: ICoN: integration using co-attention across biological networks
Source: Bioinform Adv. 2024 Nov 22;5(1):vbae182. doi: 10.1093/bioadv/vbae182 (PMC11723530; doi:10.1093/bioadv/vbae182)
Supplement: vbae182_Supplementary_Data [file vbae182_supplementary_data.zip › Manuscript__ICoN_Supplementary_Bioinformatics_Advances_Revision_1.pdf]

Journal Title Here, 2024, 1–9
doi: DOI HERE
Advance Access Publication Date: Day Month Year
Paper

ICoN: Integration using Co-attention across Biological Networks (Supplementary Document)

Nure Tasnina[1] and T. M. Murali[1]*

[1]Department of Computer Science, Virginia Tech, Blacksburg, 24061, VA, USA

*Corresponding author. murali@cs.vt.edu

FOR PUBLISHER ONLY Received on Date Month Year; revised on Date Month Year; accepted on Date Month Year

1. Supplementary notes

1.1. Effect of neighborhood sampling size on memory usage

We analyzed the effect of neighborhood sampling size on memory usage (Supplementary Figure 1). When running ICoN with the optimal hyperparameter configuration (except for neighborhood sample size) on three yeast networks, GPU memory usage increased from 1.3GB to 2.9GB as the neighborhood sample size grew from 2 to 10.

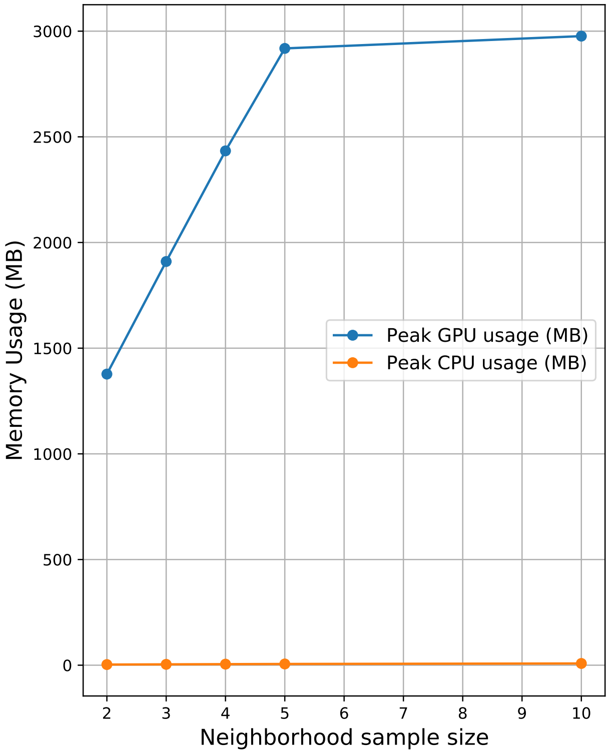

Supplementary Figure 1: **Increase in GPU memory usage with higher neighborhood sample size.**

© The Author 2024. Published by Oxford University Press. All rights reserved. For permissions, please e-mail: journals.permissions@oup.com

1.2. Degree distribution of input networks

We have reported the degree distribution of three yeast networks and four human PPI networks in Supplementary Figure 2.

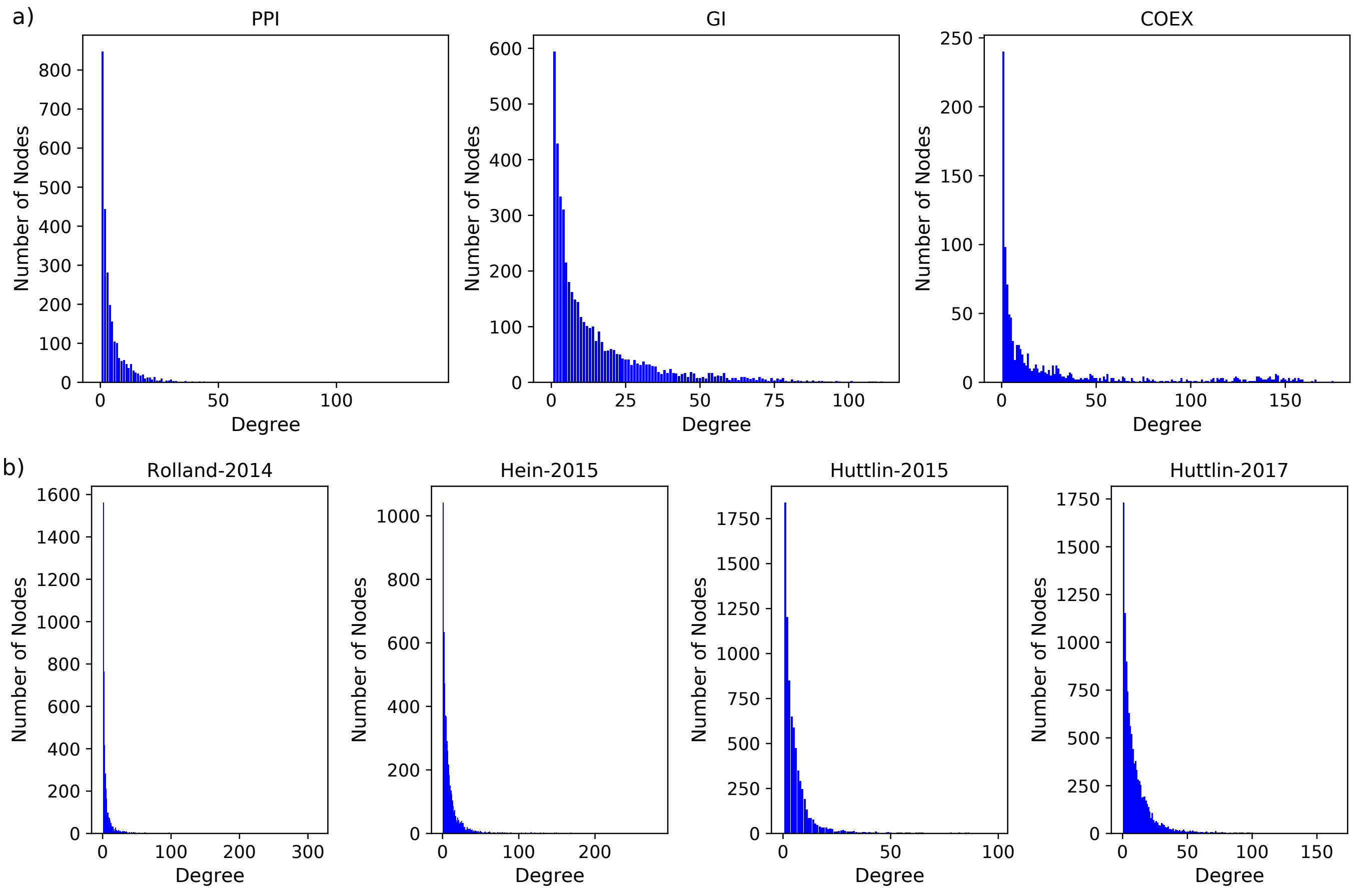

Supplementary Figure 2: **Degree distribution** of a) yeast networks b) human PPI networks.

1.3. Evaluation on the Gene Ontology benchmark

We evaluated ICoN across three downstream tasks (i.e., module detection, coannotation prediction, and function prediction) on Gene Ontology biological processes (GO BP) (Ashburner et al. (2000)) benchmark. We followed BIONIC (Forster et al., 2022) to preprocess the GO BP benchmark by excluding Inferred from Electronic Annotation (IEA) annotations due to their lower quality. Additionally, we excluded overly broad terms, i.e., those with more than 30 annotations. Consequently, the coannotation dataset for GO BP contained 4, 170 genes with 45, 015 positive pairs. The module detection and the gene function prediction benchmarks consisted of 1, 809 modules and 63 functional classes, respectively.

We observed that the embeddings generated by ICoN (i.e., by integrating three yeast networks) outperformed the individual yeast networks in all three tasks in the GO BP benchmark (Supplementary Figure 3). ICoN outranked five unsupervised network integration models in connotation prediction (Supplementary Figure 4). It performed on par with the best performing model (i.e., BERTWalk) in the module detection task (Supplementary Figure 4). However, the low scores achieved by even the best-performing models across all three tasks (AMI score of 0.23 for module detection, average precision of 0.06 for coannotation prediction, and accuracy of 0.29 for function prediction) warrant further investigation into the construction of this benchmark and its suitability for evaluation.

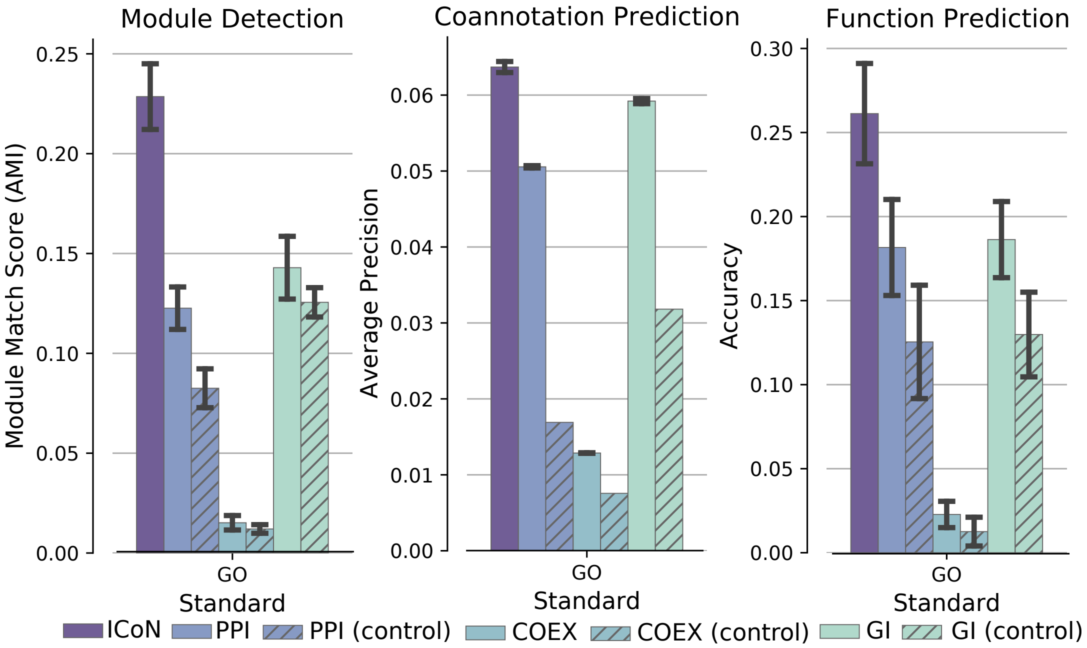

Supplementary Figure 3: **Evaluation of ICoN's performance on downstream tasks in GO BP benchmark.** Comparison of the performance of ICoN (i.e., integration of all input networks) with three individual yeast networks. Here, for each network, we have two entries e.g., "PPI" and "PPI (control)". "PPI" denotes the results for the embedding generated by ICoN with only the PPI network as input and "PPI (control)" stands for the results obtained using the adjacency matrix-based features of the network. The height of each bar indicates the average of the corresponding score and the error bar shows the standard deviation.

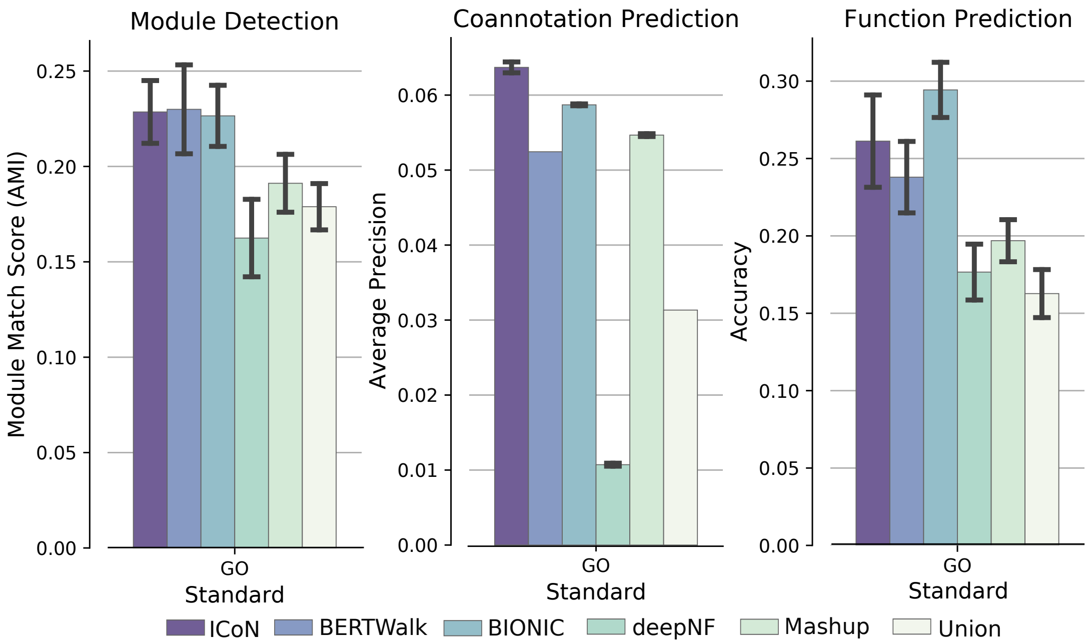

Supplementary Figure 4: **Comparison of ICoN and five unsupervised network integration methods in GO BP benchmark.** The height of each bar indicates the average of the corresponding score, and the error bar shows the standard deviation.

1.4. Hyperparameter optimization

To optimize hyperparameters for ICoN we used the same approach as BIONIC. We ran ICoN on three *S. pombe* networks, i.e., a genetic interaction network (Ryan et al. (2012)), a coexpression network (Martín et al. (2017)), and a protein–protein interaction network (Vo et al. (2016)). We evaluated the embeddings generated by different combinations of hyperparameters on gene module detection, coannotation prediction, and function prediction tasks where we utilized an *S. pombe* Gene Ontology annotation dataset (Rutherford et al. (2024), Consortium (2019)) as a gold standard. We fine-tuned five hyperparameters: (i) the number of layers in the encoder module (1, 2, or 3), (ii) the noise induction rate (0.1, 0.3, 0.5, and 0.7), (iii) the dropout rate (0.2, 0.4), (iv) the neighborhood sample size (2, 3, 4), and (v) the output dimension of the initial linear transformation layer (680, 1000). ICoN with 2 encoder layers, noise induction rate of 0.7, dropout rate of 0.4, neighborhood sample size of 4, and dimension of 1000 in linear layer achieved the highest average rank across the three tasks (Supplementary Table S1). We used these hyperparameters of ICoN henceforth. We ran BIONIC, deepNF, and Mashup with the best hyperparameters reported in the BIONIC publication. Since the BERTWalk paper did not mention hyperparameter optimization, we evaluated it on the author-provided embeddings.

1.5. Analysis of detected modules

Although Adjusted Mutual Information (AMI) provides a measure of the overall agreement between the modules detected by ICoN and the ground truth, we designed a separate experiment to assess ICoN's performance at the level of individual modules. Here, we calculated the maximum overlap (Jaccard index) of each ground truth module with predicted modules (Supplementary Figure 5a). If the Jaccard index was at least 0.5, we considered the corresponding module as having been identified. ICoN outperformed each of the individual networks by identifying the largest number of IntAct complexes and KEGG pathways (Supplementary Figure 5a). ICoN uniquely identified 87 modules in IntAct and 3 modules in KEGG that were not detected by any other networks. Conversely, ICoN missed only 30 modules in IntAct and 1 module in KEGG, that were collectively identified by other networks (Supplementary Figure 5b).

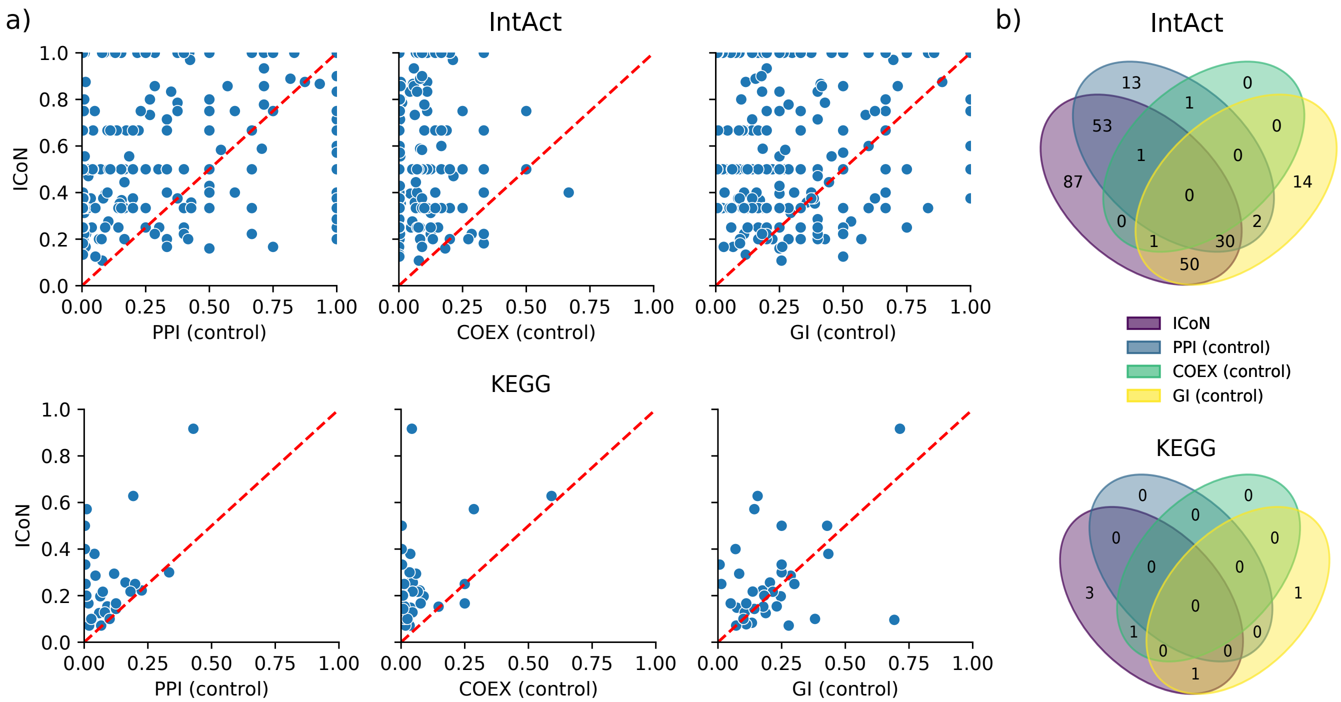

Supplementary Figure 5: **Comparison of overlap scores between known modules (i.e., protein complexes in IntAct and pathways in KEGG) and predicted modules.** a) In the scatter plots each point represents a module. The axes correspond to the overlap score, quantified by the Jaccard Index, where a score of 0 indicates that none of the module members were captured, and a score of 1.0 indicates perfect capture of the module. The diagonal line represents equivalent performance between ICoN and the given network. Points above the diagonal indicate modules where ICoN outperformed the given network, while points below the diagonal indicate modules where ICoN underperformed. b) Venn diagram of identified modules by ICoN and individual networks.

1.6. Evaluation of ICoN on combinations of networks

We sought to determine how the absence of individual input networks affects the integrated embedding in terms of performance on downstream tasks. For this analysis, we first integrated three yeast networks (i.e., PPI, COEX, GI) using ICoN. Next, we generated embeddings using ICoN but after excluding the PPI, COEX, and GI networks in turn. We denote the corresponding results as "GI+COEX", "GI+PPI", and "COEX+PPI", respectively. We observed that ICoN embeddings computed by integrating all three networks either performed better or the same as other combinations of networks in the module detection and function prediction tasks (Supplementary Figure 6). The absence of the PPI or GI networks caused a noticeable decline in performance across all three tasks in the IntAct benchmark, whereas the exclusion of the COEX network had minimal impact. Conversely, in the KEGG benchmark, removing the COEX network resulted in decreased performance across all tasks. Interestingly, for coannotation prediction in the KEGG benchmark, network combinations including COEX (i.e., GI+COEX and COEX+PPI) outperformed the integration of all three networks, highlighting the pivotal role of the COEX network.

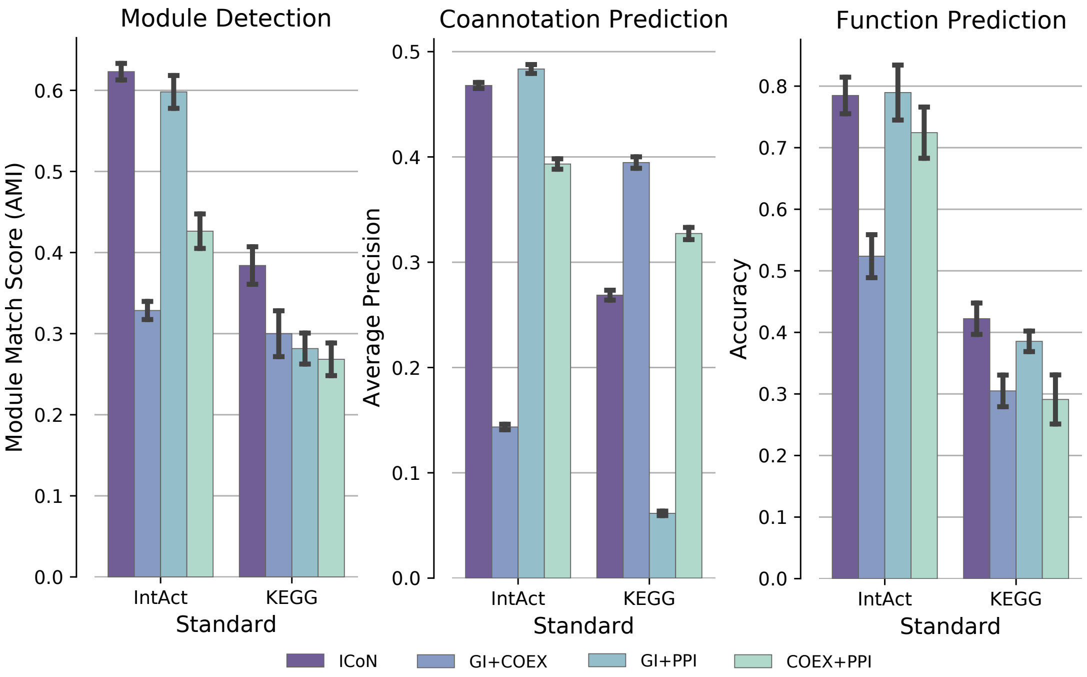

Supplementary Figure 6: **Comparison of performance across different combinations of input networks.**

2. Supplementary figures

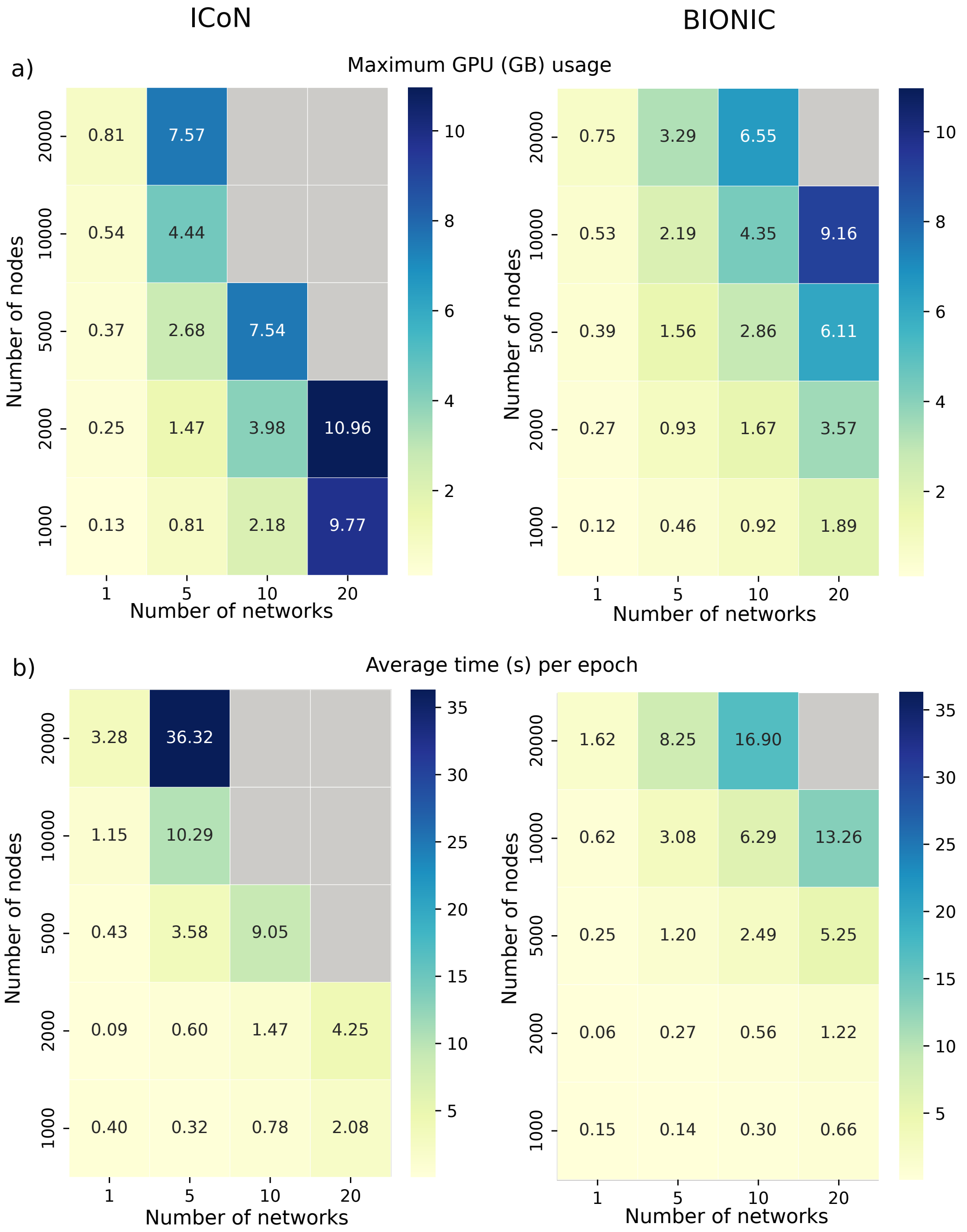

Supplementary Figure 7: **Comparison of scalability between ICoN and BIONIC**. Each model was run on NVIDIA A2 GPU with a 3 GHz AMD EPYC 7313 16-Core Processor and 78 GB of RAM.

References

Ashburner, M., Ball, C. A., Blake, J. A., Botstein, D., Butler, H., Cherry, J. M., Davis, A. P., Dolinski, K., Dwight, S. S., Eppig, J. T., et al. (2000). Gene ontology: tool for the unification of biology. *Nature Genetics*, 25(1):25–29.

Consortium, G. O. (2019). The gene ontology resource: 20 years and still going strong. *Nucleic acids research*, 47(D1):D330–D338.

Forster, D. T., Li, S. C., Yashiroda, Y., Yoshimura, M., Li, Z., Isuhuaylas, L. A. V., Itto-Nakama, K., Yamanaka, D., Ohya, Y., Osada, H., et al. (2022). BIONIC: biological network integration using convolutions. *Nature Methods*, 19(10):1250–1261.

Martín, R., Portantier, M., Chica, N., Nyquist-Andersen, M., Mata, J., and Lopez-Aviles, S. (2017). A PP2A-B55-mediated crosstalk between TORC1 and TORC2 regulates the differentiation response in fission yeast. *Current Biology*, 27(2):175–188.

Rutherford, K. M., Lera-Ramírez, M., and Wood, V. (2024). Pombase: a global core biodata resource—growth, collaboration, and sustainability. *Genetics*, 227(1):iyae007.

Ryan, C. J., Roguev, A., Patrick, K., Xu, J., Jahari, H., Tong, Z., Beltrao, P., Shales, M., Qu, H., Collins, S. R., et al. (2012). Hierarchical modularity and the evolution of genetic interactomes across species. *Molecular Cell*, 46(5):691–704.

Vo, T. V., Das, J., Meyer, M. J., Cordero, N. A., Akturk, N., Wei, X., Fair, B. J., Degatano, A. G., Fragoza, R., Liu, L. G., et al. (2016). A proteome-wide fission yeast interactome reveals network evolution principles from yeasts to human. *Cell*, 164(1):310–323.
